# Lipoprotein(a) as a Risk Factor for Myocardial Infarction, Cardiovascular, and All-Cause Mortality in Patients with Type 2 Diabetes Mellitus

**DOI:** 10.3390/diagnostics16101520

**Published:** 2026-05-18

**Authors:** Jerneja Čuješ, Vojko Kanič, Petra Povalej Bržan, David Šuran

**Affiliations:** 1 University Department of Cardiology and Angiology, University Medical Center Maribor, Ljubljanska Ulica 5, 2000 Maribor, Slovenia; vojko.kanic@guest.arnes.si (V.K.); david.suran@ukc-mb.si (D.Š.); 2 Faculty of Medicine, University of Maribor, Taborska Ulica 8, 2000 Maribor, Slovenia; petra.povalej@um.si; 3 Faculty of Electrical Engineering and Computer Science, University of Maribor, Koroška Cesta 46, 2000 Maribor, Slovenia

**Keywords:** lipoprotein(a), risk factor, type 2 diabetes mellitus, myocardial infarction, all-cause mortality, cardiovascular mortality

## Abstract

**Background**: Lipoprotein(a) (Lp(a)) is a genetically determined lipoprotein associated with atherosclerotic cardiovascular disease (ASCVD). Its prognostic role in type 2 diabetes mellitus (T2DM) remains unclear. We aimed to evaluate the association of Lp(a) with first myocardial infarction (MI), cardiovascular (CV) mortality, and all-cause mortality, with a focus on sex differences. **Methods**: We retrospectively analysed patients with T2DM and no prior MI who were hospitalised between 2000 and 2018 and had baseline Lp(a) measurements. Patients were followed until MI, death, or the end of 2023. Lp(a) was categorised as ≤50, 51–90, and >90 mg/dL. Cox proportional hazards models were adjusted for low-density lipoprotein cholesterol, arterial hypertension, and estimated glomerular filtration rate < 60 mL/min/1.73 m^2^, with attained age used as the time scale. **Results**: A total of 2967 patients (37.5% women) were included. During follow-up, 12.5% of patients experienced MI, 36.4% died from CV causes, and 68.8% died from any cause. A significant interaction between Lp(a) and sex was observed for MI. In sex-specific analyses, Lp(a) > 50 mg/dL was associated with a higher risk of first MI in women (51–90 mg/dL: HR 1.79, 95% CI 1.07–2.99; >90 mg/dL: HR 2.67, 95% CI 1.66–4.32), whereas no significant association was observed in men. Elevated Lp(a) ≥ 50 mg/dL was also associated with higher CV mortality overall (51–90 mg/dL: HR 1.42, 95% CI 1.07–1.89; >90 mg/dL: HR 1.38, 95% CI 1.01–1.91), without a significant interaction with sex. No significant associations were observed for all-cause mortality. **Conclusions**: In patients with T2DM, elevated Lp(a) > 50 mg/dL was associated with increased risk of first MI, predominantly in women, and with higher CV mortality overall. Lp(a) may serve as a valuable marker for CV risk stratification in this population.

## 1. Introduction

Type 2 diabetes mellitus (T2DM) is a major global health concern and a leading risk factor for atherosclerotic cardiovascular disease (ASCVD), particularly myocardial infarction (MI). Among individuals with T2DM, MI remains the leading cause of morbidity and mortality [1].

Lipoprotein(a) (Lp(a)) is a lipoprotein particle with pro-atherogenic, pro-inflammatory, and potentially prothrombotic properties [2]. Current evidence indicates that its concentration is largely genetically determined and largely independent of diet and lifestyle modifications [2,3,4]. In men, Lp(a) levels remain relatively stable throughout life, whereas a modest increase has been observed in women after menopause [2,5,6,7].

Among currently available therapies for hypercholesterolemia, only proprotein convertase subtilisin/kexin type 9 (PCSK9) inhibitors reduce Lp(a) levels by approximately 25% [8,9]. Novel Lp(a)-targeted therapies are under investigation, including the antisense oligonucleotide pelacarsen and small interfering ribonucleic acid [10,11].

In the general population, extensive epidemiological studies and Mendelian randomisation analyses have established Lp(a) as a causal risk factor for ASCVD across multiple vascular beds, including the coronary, cerebrovascular, and peripheral arteries [4,5,12,13,14]. However, data on the role of Lp(a) in patients with T2DM remain limited and heterogeneous [15,16,17,18,19,20,21,22]. Some studies have reported an association between elevated Lp(a) levels and increased risk of MI or adverse outcomes in patients with T2DM [15,16,23], whereas others have suggested that this association may be attenuated or absent in this population [17,18,22]. In particular, evidence regarding the relationship between Lp(a) and cardiovascular (CV) or all-cause mortality in T2DM, as well as the potential modifying effects of sex, remains limited and inconsistent [17,19,21].

To address knowledge gaps, the present study aimed to evaluate the association between Lp(a) levels and the risk of first MI, CV mortality, and all-cause mortality among patients with T2DM, and to examine potential sex differences.

## 2. Materials and Methods

### 2.1. Patients

Based on the Tenth Revision of the International Classification of Diseases (ICD-10), we included patients with T2DM and no history of MI who were hospitalised at the Clinic for Internal Medicine, University Medical Center Maribor, between 2000 and 2018, and had Lp(a) measurements available at admission. Most Lp(a) measurements were obtained as part of the routine admission laboratory protocol. A smaller proportion was measured at the attending physician’s discretion to provide a more detailed assessment of CV risk.

Patients treated with PCSK9 inhibitors were excluded because of their known effects on Lp(a) levels [1,8]. Additional exclusion criteria included type 1 diabetes mellitus, incomplete medical documentation, and advanced chronic kidney disease with an estimated glomerular filtration rate (eGFR) below 20 mL/min.

The study protocol was approved by the National Medical Ethics Committee of the Republic of Slovenia (0120-523/2023-2711-4; 1 March 2023). The retrospective data analysis was conducted in accordance with the ethical principles of the Declaration of Helsinki.

#### 2.1.1. Clinical Variables

Data on all clinical variables, including clinical diagnoses and medication use, were obtained from the institutional electronic hospital database at the time of inclusion. Medication data included lipid-lowering therapy, as well as antidiabetic, antiplatelet, and anticoagulant treatments at hospital discharge or at baseline hospitalisation. Antidiabetic therapy was categorised as oral antidiabetic agents or insulin therapy at baseline hospitalisation. Arterial hypertension was defined using ICD-10 codes recorded at the time of inclusion. Kidney function was assessed using the eGFR, calculated with the Modification of Diet in Renal Disease (MDRD) formula [24].

#### 2.1.2. Laboratory Tests

Blood samples for all laboratory parameters were collected and analysed on the day of hospital admission. All serum Lp(a) levels were determined using a fully automated particle-enhanced immunonephelometric assay (Siemens ProSpec, Siemens Healthineers, Erlangen, Germany) and reported in mass units (mg/dL). According to the manufacturer, the assay has a coefficient of variation of 6.0% at 0.288 g/L and 4.7% at 0.698 g/L, with a recommended maximum allowable coefficient of variation of <10% (including lot-to-lot, operator, and inter-analyser variability), which aligns with our laboratory’s performance.

Low-density lipoprotein cholesterol (LDL-C) was measured by a homogeneous direct method (Siemens Dimension Vista, Siemens Healthineers, Erlangen, Germany).

#### 2.1.3. Follow-Up and Outcomes

Patients were followed from their baseline hospitalisation, when Lp(a) and other laboratory parameters were measured (2000–2018), until the occurrence of an event—MI, CV death, or all-cause death—or until the end of 2023, whichever occurred first. Information on MI hospitalisations during follow-up was obtained from the institutional electronic hospital database and regional hospitals.

For patients who died during the observational period, the date of death and ICD-10 codes indicating the principal cause of death were obtained from the National Institute of Public Health of Slovenia (National Mortality Database).

First MI was defined as the first hospitalisation with a principal ICD-10 code of I21 or I22, or as an event in which one of these codes was recorded as the principal cause of death in the National Mortality Database.

CV death was defined as death with a primary ICD-10 code from I00 to I99.

### 2.2. Statistical Analysis

Only patients with complete data for all variables included in the regression models were included in the analysis; therefore, no imputation of missing data was performed.

All analyses were conducted in the R statistical software (R version 4.5.2 (31 October 2025 ucrt), R Foundation for Statistical Computing, Vienna, Austria), primarily using the survival package. Continuous variables are presented as medians with interquartile ranges (IQRs). Normality was assessed using the Anderson–Darling test, complemented by visual inspection of histograms and Q-Q plots. Categorical variables are presented as counts and percentages.

Baseline characteristics were summarised overall and stratified by sex. Continuous variables were compared using the Wilcoxon rank-sum test, and categorical variables using Pearson’s chi-squared test. Standardised mean differences (SMDs) were calculated to quantify between-group differences independently of sample size, with values of 0.1, 0.3, and 0.5 interpreted as small, moderate, and large differences, respectively.

Lp(a) was analysed as a categorical variable, grouped into three categories: ≤50 mg/dL, 51–90 mg/dL, and >90 mg/dL, with the ≤50 mg/dL group serving as the reference category. These thresholds were selected based on clinically relevant cut-offs reported in previous studies and guideline recommendations [5,13,25]. The 50 mg/dL threshold is widely used to define elevated Lp(a) levels. By contrast, higher thresholds (e.g., 90 mg/dL) have been used in observational studies and ongoing clinical trials to explore potential gradients of CV risk [10,12,13].

Time-to-event analyses for MI, CV mortality, and all-cause mortality were conducted using Kaplan–Meier methods and Cox proportional hazards (PH) regression models. For all survival analyses, attained age was used as the time scale. Participants entered the risk set at their age at baseline hospitalisation (defined as the time point of the first Lp(a) measurement). They were followed until the event of interest, death, or the end of follow-up. This approach provides more robust control for age, a dominant risk factor, than including age solely as a covariate in the model. All variables were assessed at baseline hospitalisation.

Multivariable Cox PH models were used to assess associations between Lp(a) categories and outcomes, adjusting for sex, reduced kidney function (eGFR < 60 mL/min/1.73 m^2^), arterial hypertension, and LDL-C. To mitigate potential collinearity between Lp(a) and LDL-C, LDL-C values were corrected by subtracting an estimated cholesterol fraction of Lp(a), assumed to represent approximately 30% of total Lp(a) mass [26], as used in some large epidemiological studies [13,27]. This approach offers a pragmatic approximation of Lp(a)-free LDL-C, though it does not account for interindividual variability in Lp(a) composition.

The PH assumption was assessed using Schoenfeld residuals and graphical diagnostics. When violations were detected, appropriate corrective strategies were applied, including stratification and time-dependent modelling. Covariates for which a constant hazard ratio (HR) was not required for interpretation were handled by stratification, allowing baseline hazards to differ across strata. When sex violated the PH assumption, it was modelled as a time-dependent effect using an interaction with log(time), allowing its effect to vary with attained age while sex itself remained a fixed baseline characteristic.

Potential effect modification by sex was formally assessed by including an interaction term between Lp(a) categories and sex and evaluating it using a likelihood ratio. When a statistically significant interaction was observed, separate Cox PH models were fitted for women and men, allowing all regression coefficients to be estimated independently within each sex and providing clinically interpretable, sex-specific HR estimates.

For all-cause mortality, reduced kidney function, and hypertension, the PH assumption was violated, so they were included as stratification variables in the Cox model.

HRs are reported with 95% confidence intervals (CIs) and interpreted at the same attained age. A two-sided *p*-value < 0.05 was considered statistically significant.

## 3. Results

### 3.1. Baseline Statistics

A total of 2967 patients with T2DM and no prior MI were included in the analysis. The median age at inclusion was 68 years (IQR 60–74), and 1113 patients (37.5%) were women. At inclusion, corresponding to the baseline hospitalisation when Lp(a) was measured, 29% were treated with insulin and 67% received oral antidiabetic therapy. Statin therapy was used by 63% of patients, antiplatelet therapy by 60%, and anticoagulant therapy by 14%. Median follow-up durations were 15.1 years (IQR 11.5–18.5) for MI, 12.7 years (IQR 6.8–17.1) for CV mortality, and 9.1 years (IQR 3.7–14.0) for all-cause mortality.

The median Lp(a) concentration was 12 mg/dL (IQR 5–35). Patients were categorised by Lp(a) levels: ≤50 mg/dL (83.4%), 51–90 mg/dL (9.3%), and >90 mg/dL (7.3%). During follow-up, 12.5% of patients in the overall cohort were hospitalised for MI, 36.4% died from CV causes, and 68.8% died from any cause. The median eGFR was 65.2 mL/min/1.73 m^2^ (IQR 51.3–79.3), and 40.1% of patients had an eGFR < 60 mL/min/1.73 m^2^. Detailed baseline characteristics are shown in Table 1. Baseline characteristics stratified by sex are presented in Table S1, where the largest differences were observed in age and kidney function, whereas most other characteristics were comparable between the sexes.

### 3.2. Association Between Lp(a) Intervals and MI

**Figure 1:**
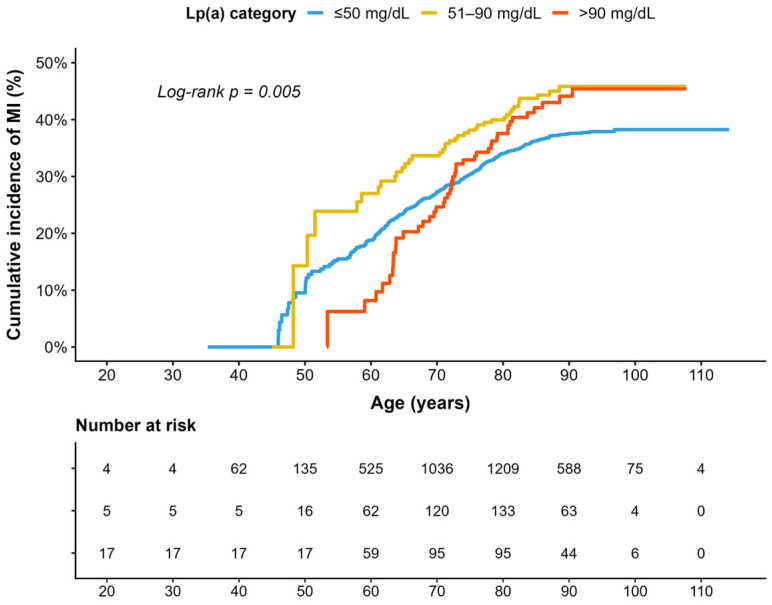
Cumulative incidence of MI by Lp(a) category. Kaplan–Meier curves show the cumulative incidence of MI by Lp(a) category. Attained age was used as the time scale. A significant difference across groups was observed (log-rank *p* = 0.0018). Curves show partial overlap and crossing, particularly between the two higher Lp(a) categories (51–90 mg/dL and >90 mg/dL), with no clear separation between them. MI—myocardial infarction; Lp(a)—lipoprotein(a).

Kaplan–Meier analysis showed significantly different MI-free survival across Lp(a) categories (log-rank *p* = 0.0018). The cumulative incidence curves overlapped and crossed between groups, with no consistent ordering across the age range. The two higher Lp(a) categories followed broadly similar trajectories, particularly at older ages, with no clear separation between them (Figure 1).

In the multivariable Cox PH model with an interaction between Lp(a) and sex (Table 2), elevated Lp(a) levels were significantly associated with increased MI risk in a sex-dependent manner. Compared with the reference category (≤50 mg/dL), HRs for women were 1.83 for Lp(a) 51–90 mg/dL and 2.80 for Lp(a) > 90 mg/dL. The interaction terms indicated that the association was attenuated in men (51–90 mg/dL × male: HR 0.47; >90 mg/dL × male: HR 0.49), consistent with a sex-specific effect of Lp(a) on MI risk.

**Figure 2:**
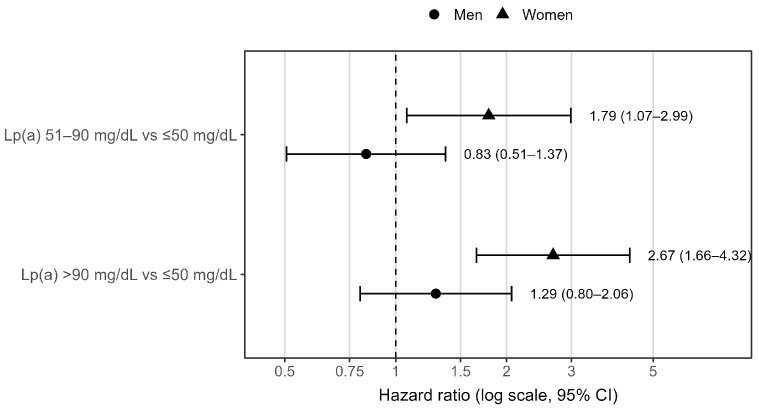
Sex-specific hazard ratios for MI by Lp(a) category. HRs and 95% CIs were estimated using sex-specific Cox PH models and are shown on a logarithmic *x*-axis. The reference category was Lp(a) ≤ 50 mg/dL. Models were adjusted for LDL-C, arterial hypertension, and eGFR < 60 mL/min/1.73 m^2^. The interaction between Lp(a) category and sex was statistically significant (*p* for interaction [Lp(a) × sex] = 0.030). The dashed line denotes HR = 1, indicating no difference in hazard compared with the reference Lp(a) category. Lp(a)—lipoprotein(a); LDL-C—low-density lipoprotein cholesterol; MI—myocardial infarction; HR—hazard ratio; CI—confidence interval.

To further explore the observed sex-specific interaction, separate Cox PH models were fitted for men and women. Among women, elevated Lp(a) levels were strongly associated with MI (Figure 2). Compared with women with Lp(a) ≤ 50 mg/dL, those with Lp(a) 51–90 mg/dL had a significantly higher risk of MI (*p* = 0.026), while those with Lp(a) > 90 mg/dL had an approximately 2.7-fold higher risk (*p* < 0.001). In contrast, no statistically significant association between Lp(a) and MI was observed in men (Table 2).

### 3.3. Association Between Lp(a) and CV/All-Cause Mortality

**Figure 3:**
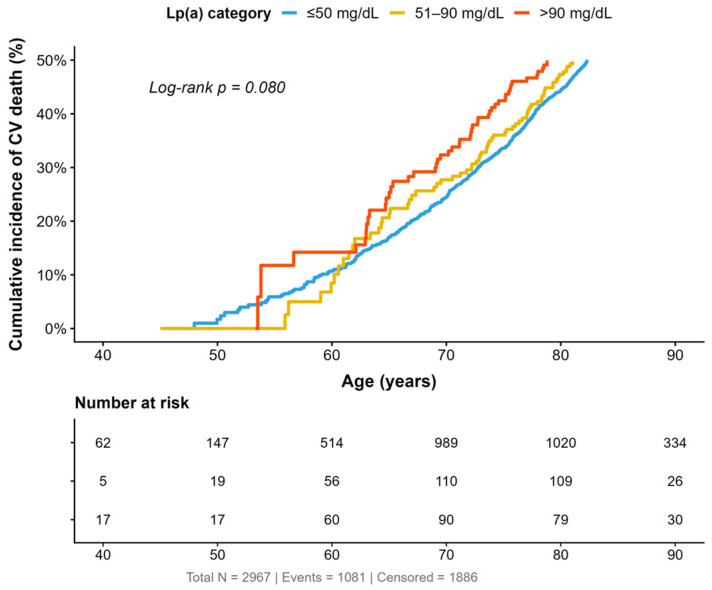
Cumulative incidence of CV death by Lp(a) categories. Lp(a)—lipoprotein(a); CV—cardiovascular.

Kaplan–Meier curves using attained age as the time scale showed no statistically significant differences in CV mortality across Lp(a) categories (log-rank *p* = 0.080). Although the >90 mg/dL group had a slightly higher cumulative incidence at intermediate ages, the curves overlapped substantially throughout follow-up (Figure 3).

In the multivariable Cox PH model, elevated Lp(a) concentrations were associated with a higher risk of CV mortality. Compared with the reference category (Lp(a) ≤ 50 mg/dL), patients with Lp(a) levels of 51–90 mg/dL had a 42% higher risk of CV death, while those with Lp(a) > 90 mg/dL had a 38% higher risk (Table 3).

In the sex-interaction analyses, the association between Lp(a) and CV mortality appeared attenuated in men; however, the interaction terms were not statistically significant (Lp(a) 51–90 × male: HR 0.70; Lp(a) > 90 × male: HR 0.74), indicating no statistically significant effect modification by sex (Table 3).

In multivariable Cox PH models for all-cause mortality, Lp(a) concentrations were not independently associated with risk (51–90 mg/dL: HR 1.06, 95% CI 0.85–1.34, *p* = 0.592; >90 mg/dL: HR 1.04, 95% CI 0.80–1.34, *p* = 0.775). There was no evidence of significant effect modification by sex (interaction terms: 51–90 mg/dL × male: HR 0.84, 95% CI 0.62–1.15, *p* = 0.277; >90 mg/dL × male: HR 0.81, 95% CI 0.57–1.15, *p* = 0.242), indicating no statistically significant interaction between Lp(a) and sex (Table S2).

The discrepancy between Kaplan–Meier and Cox model results likely reflects adjustment for confounding variables in the multivariable analysis, which is not accounted for in unadjusted Kaplan–Meier estimates.

## 4. Discussion

We conducted a retrospective observational cohort study of patients with T2DM to examine the association between Lp(a) levels and the first occurrence of MI, as well as CV and all-cause mortality. Elevated Lp(a) levels (>50 mg/dL) were associated with an increased risk of MI, predominantly in women. Higher Lp(a) levels were also associated with increased CV mortality in the overall cohort, although the effect estimates were numerically higher in women. No significant associations were observed between Lp(a) levels and all-cause mortality.

### 4.1. Association Between Lp(a) Levels and MI

Meta-analyses of epidemiological and Mendelian randomisation studies have confirmed a causal relationship between Lp(a) and MI in the general population [4,12,13]. Kamstrup et al. (2008) demonstrated a three- to fourfold increased risk of MI among individuals with Lp(a) levels above the 95th percentile in the general population [13]. However, findings from studies conducted specifically in patients with T2DM and MI have been inconsistent [15,16,17,20,27,28,29].

Mader et al. (prospective cohort, n = 1200, age <65, patients with coronary artery disease) reported that elevated Lp(a) was associated with a higher risk of CV events only in non-diabetic patients, suggesting that T2DM may attenuate its prognostic relevance [17]. In contrast, a prospective study published in 2021 found that although patients with T2DM had lower Lp(a) levels than the non-diabetic population, elevated Lp(a) remained associated with early-stage coronary artery disease [15]. Similarly, two large retrospective registry datasets, including patients with and without diabetes, showed that elevated Lp(a) was associated with an increased incidence of MI across both populations, with a particularly higher risk in people with diabetes [27,28].

In our cohort, Lp(a) > 50 mg/dL was associated with an increased risk of MI in women (HR 1.83, *p* = 0.020), with a further increase in risk at Lp(a) levels > 90 mg/dL (HR 2.80, *p* < 0.001). These findings are consistent only with some previous reports in the general population, which showed stronger associations between elevated Lp(a) and first or recurrent MI in women [30,31,32]. In contrast, Kamstrup et al. reported that, in the general population, elevated Lp(a) levels were associated with an increased risk of MI and ischaemic heart disease in both women and men [13]. Data on sex-specific differences in the association between Lp(a) levels and MI in diabetic patients remain limited. However, some studies have similarly suggested that elevated Lp(a) may be associated with extensive coronary calcification and higher risk of non-fatal MI predominantly in women with T2DM, but not in men or the non-diabetic population [33,34].

The stronger association between elevated Lp(a) levels and MI observed in women may partly be explained by postmenopausal increases in Lp(a) levels and by changes in the overall CV risk profile that may enhance the atherogenic impact of Lp(a) [6,21,30,31,33]. Postmenopausal increases may contribute to higher CV risk through the pro-atherogenic and pro-thrombotic effects of Lp(a), which may interact with metabolic alterations associated with T2DM and hormonal changes in women [21,33,35,36].

Although our cohort consisted mainly of elderly individuals, and thus most women were likely postmenopausal, the increased CV risk associated with T2DM in women has also been reported in premenopausal populations [35]. T2DM is thought to attenuate the protective effects of female sex hormones, thereby diminishing the usual CV advantage observed in women before menopause [35,37]. This mechanism may also partly contribute to the more pronounced prognostic role of Lp(a) observed in women in our study. The observed sex-specific associations should be considered hypothesis-generating and require confirmation in independent cohorts.

### 4.2. Association Between Lp(a) Level and CV and All-Cause Mortality

Studies investigating the association between Lp(a) and CV or all-cause mortality have reported inconsistent results in both the general population and patients with T2DM [12,18,19,20,21,27,28,30,31,32,38,39]. Some studies have reported a positive association between elevated Lp(a) levels and CV or all-cause mortality only in non-diabetic individuals [17,20,39]. Other studies found a positive association with CV mortality in both diabetic and non-diabetic groups [28,33], but not with all-cause mortality [27]. In contrast, Fras et al. reported significantly higher CV and all-cause mortality among patients with diabetes and Lp(a) > 50 mg/dL, whereas no such association was observed in non-diabetic individuals [19]. Our findings align with some previous reports, showing that Lp(a) levels > 50 mg/dL, after adjustment for potential confounders, independently predict CV mortality in patients with T2DM. Our interaction analysis suggested a non-significant trend towards a stronger effect in women.

Our study observed a slight, non-significant trend towards increased all-cause mortality in the group with Lp(a) > 50 mg/dL. In the sex-interaction analysis, women showed a non-significant trend towards higher all-cause mortality, mirroring the pattern seen for CV mortality. Unlike some large population studies that found a link between elevated Lp(a) and all-cause mortality [12,39], we did not find a statistically significant association. This difference may be due to the high baseline CV risk in T2DM patients, where multiple competing risk factors could diminish Lp(a)’s impact on overall mortality.

Data on the potential differential impact of sex on this association are scarce. Our results align with some population-based studies in the general [32] and diabetic populations [21,33], indicating that women, particularly postmenopausal women, may experience a greater Lp(a)-associated increase in relative CV mortality risk [21,32]. However, in the absence of statistically significant sex interaction effects, our observations of higher CV mortality risk in women should be regarded as hypothesis-generating rather than confirmatory.

### 4.3. Strengths and Limitations of the Study

This study has several strengths. It includes a relatively large cohort of patients with T2DM and comprehensive clinical data, enabling robust statistical analyses. It is also among the few studies to examine sex-specific associations of Lp(a) with MI and CV/all-cause mortality in patients with T2DM. Our findings suggest that Lp(a) may be a useful marker for CV risk stratification in selected subgroups of patients with T2DM, particularly women, and support further investigation of Lp(a)-lowering therapies in this high-risk population [10,11].

Several limitations should also be acknowledged. Our cohort consisted predominantly of elderly individuals (median age 67.9; IQR 60.2–74.7); therefore, our results may not be entirely applicable to the general population. In addition, because the selection process involved hospitalised patients, our cohort had a higher overall disease burden, and our conclusions cannot be fully generalisable to the broader population. Furthermore, the analyses were based on ICD-10 diagnoses recorded at baseline by the discharging physician. A similar limitation applies to the definition of CV death, which is inherently challenging, particularly for out-of-hospital deaths, as it relies on the cause of death recorded by the certifying physician. Therefore, the data on CV mortality should be interpreted as informative rather than definitive.

Lp(a) was measured in mass units (mg/dL); molar units (nmol/L) may better reflect apo(a) isoform variability, although both approaches are accepted when used consistently within a study [5,9].

LDL-C was not an independent predictor of MI in our models, which contrasts with extensive evidence supporting its role in ASCVD [9,40,41]. This may be explained by the use of baseline measurements only, which do not reflect cumulative lifetime LDL-C exposure or the impact of lipid-lowering therapy during follow-up, as well as by the partial correlation of LDL-C with intensive lipid-lowering treatment within the cohort.

Residual confounding cannot be ruled out because of the retrospective design and the incomplete availability of key covariates (e.g., HbA1c, body mass index, diabetes duration, statin intensity, socioeconomic factors). However, the findings are biologically plausible given the genetic stability of Lp(a), and the sex-specific results should be considered hypothesis-generating [2].

Additional sensitivity analyses (e.g., alternative model specifications) were not conducted and could further support the robustness of the findings.

We acknowledge that death is a competing risk for MI. As our primary objective was to estimate cause-specific HRs, we used Cox regression; however, competing risk models could provide complementary insights into cumulative incidence.

## 5. Conclusions

In patients with T2DM without prior MI, elevated Lp(a) levels above 50 mg/dL were independently associated with an increased risk of first MI, particularly in women. In addition, elevated Lp(a) levels > 50 mg/dL were associated with higher CV mortality in the overall cohort, although effect estimates were numerically higher in women. No significant association was observed between Lp(a) and all-cause mortality.

These findings suggest that Lp(a) may serve as a useful marker for CV risk stratification in patients with T2DM. Further studies are needed to confirm the observed sex-related differences and to evaluate the role of emerging targeted Lp(a)-lowering therapies in this high-risk population.

## Figures and Tables

**Table 1:** Baseline clinical characteristics by Lp(a) categories.

Variable	Overall Cohort	Lp(a) ≤ 50 mg/dL	Lp(a) 51–90 mg/dL	Lp(a) > 90 mg/dL
Number of patients	2967	2474	275	218
Age, years ^1^	67.9 (60.2–74.7)	68.1 (60.3–74.8)	67.8 (60.6–74.0)	66.1 (59.3–73.6)
Sex—female ^2^	1113 (37.5%)	905 (36.6%)	112 (40.7%)	96 (44.0%)
MI ^2^	372 (12.5%)	292 (11.8%)	37 (13.5%)	43 (19.7%)
CV mortality ^2^	1081 (36.4%)	884 (35.7%)	110 (40.0%)	87 (39.9%)
All-cause mortality ^2^	2042 (68.8%)	1706 (69.0%)	190 (69.1%)	146 (67.0%)
Arterial hypertension ^2^	2109 (71.1%)	1752 (70.8%)	195 (70.9%)	162 (74.3%)
LDL-C, mmol/L ^1^	2.4 (1.8–3.2)	2.5 (1.9–3.3)	2.2 (1.6–3.0)	1.9 (1.5–2.5)
eGFR < 60 mL/min/1.73 m^2 2^	1190 (40.1%)	1003 (40.5%)	98 (35.6%)	89 (40.8%)

^1^ Median (Q1–Q3) for continuous variables; ^2^ n (%) for categorical variables; Lp(a)—lipoprotein(a); LDL-C—low-density lipoprotein cholesterol; eGFR—estimated glomerular filtration rate; MI—myocardial infarction; CV—cardiovascular.

**Table 2:** Multivariable Cox PH model demonstrating the association of Lp(a) with MI, including sex interaction.

Variable	HR (95% CI)	*p*-Value
Lp(a) 51–90 mg/dL (vs. ≤50 mg/dL)	1.83 (1.10–3.06)	0.020
Lp(a) > 90 mg/dL (vs. ≤50 mg/dL)	2.80 (1.74–4.51)	<0.001
Lp(a) 51–90 × male sex	0.47 (0.23–0.95)	0.037
Lp(a) > 90 × male sex	0.49 (0.25–0.97)	0.039
eGFR < 60 mL/min/1.73 m^2^	1.20 (0.95–1.51)	0.124
Hypertension	1.23 (0.97–1.57)	0.089
LDL-C (per 1 mg/dL increase)	1.08 (0.98–1.19)	0.141

Age was used as the time scale. Sex violated the PH assumption and was therefore modelled as a time-dependent covariate (tt(male01) = male01 × log(t)); consequently, a single HR for sex is not presented. HRs for Lp(a) categories are adjusted for all shown covariates and are interpreted at the same attained age. Reference category for Lp(a): ≤50 mg/dL; reference sex: female. The HRs for Lp(a) categories, therefore, correspond to women, while the interaction terms (Lp(a) × male) indicate how these associations differ between men and women. Lp(a)—lipoprotein(a); LDL-C—low-density lipoprotein cholesterol; eGFR—estimated glomerular filtration rate; MI—myocardial infarction; HR—hazard ratio; CI—confidence interval; PH—proportional hazards.

**Table 3:** Multivariable Cox PH model demonstrating the association of Lp(a) with CV mortality, including sex interaction.

Variable	HR (95% CI)	*p*-Value
Lp(a) 51–90 mg/dL	1.42 (1.07–1.89)	0.014
Lp(a) > 90 mg/dL	1.38 (1.01–1.91)	0.046
Lp(a) 51–90 × male sex	0.70 (0.47–1.05)	0.083
Lp(a) > 90 × male sex	0.74 (0.47–1.16)	0.194
eGFR < 60 mL/min/1.73 m^2^	1.69 (1.49–1.93)	<0.001
Hypertension	0.98 (0.86–1.12)	0.766
LDL-C (per 1 mg/dL increase)	0.96 (0.90–1.02)	0.207

Sex violated the PH assumption and was therefore modelled as a time-dependent covariate; a single HR for sex is not presented. HRs for Lp(a) categories are adjusted for all shown covariates and interpreted at the same attained age. Reference category for Lp(a): ≤50 mg/dL; reference sex: female. Lp(a)—lipoprotein(a); LDL-C—low-density lipoprotein cholesterol; eGFR—estimated glomerular filtration rate; PH—proportional hazards.

## Data Availability

The data that support the findings of this study are available from the corresponding author upon a reasonable request. The data are not publicly available due to ethical concerns.

## Supplementary Materials

The following supporting information can be downloaded at: https://www.mdpi.com/article/10.3390/diagnostics16101520/s1, Table S1: Baseline characteristics of the study population stratified by sex at study entry; Table S2: Multivariable Cox PH model demonstrating the association of Lp(a) with all-cause mortality, including sex interaction.

## Abbreviations

The following abbreviations are used in this manuscript:

ASCVD	Atherosclerotic cardiovascular disease
MI	Myocardial infarction
CI	Confidence interval
CV	Cardiovascular
eGFR	Estimated glomerular filtration rate
HR	Hazard ratio
ICD-10	International Classification of Diseases
IQR	Interquartile range
LDL-C	Low-density lipoprotein cholesterol
Lp(a)	Lipoprotein(a)
MDRD	Modification of the diet in renal disease formula
PCSK9	Proprotein Convertase Subtilisin/Kexin type 9
PH	Proportional hazard
SMD	Standardized mean difference
T2DM	Type 2 Diabetes Mellitus
