# Supplementary material for: Lipoprotein(a) as a Risk Factor for Myocardial Infarction, Cardiovascular, and All-Cause Mortality in Patients with Type 2 Diabetes Mellitus
Source: Diagnostics (Basel). 2026 May 18;16(10):1520. doi: 10.3390/diagnostics16101520 (PMC13205150; doi:10.3390/diagnostics16101520)
Supplement: Supplementary file 1 [file diagnostics-16-01520-s001.zip › diagnostics-4262806-supplementary.pdf]

diagnostics

MDPI

Table S1. Baseline characteristics of the study population stratified by sex at study entry.

Characteristic	Overall N = 2,967	Female N = 1,113	Male N = 1,854	SMD
Age, years	68.00 [60.00, 74.00]	71.00 [64.00, 77.00]	65.00 [58.00, 72.00]	0.577
Lp(a)	12.00 [5.00, 35.00]	14.00 [6.00, 39.00]	12.00 [4.00, 33.75]	0.112
Lp(a) category				0.093
≤50 mg/dL	2474 (83.4%)	905 (81.3%)	1569 (84.6%)	
51–90 mg/dL	275 (9.3%)	112 (10.1%)	163 (8.8%)	
>90 mg/dL	218 (7.3%)	96 (8.6%)	122 (6.6%)	
LDL-C, mmol/L	2.63 [2.05, 3.39]	2.70 [2.09, 3.50]	2.60 [2.01, 3.30]	0.086
eGFR <60 mL/min/1.73 m^2	1190 (40.1%)	633 (56.9%)	557 (30.0%)	0.562
Arterial hypertension	2109 (71.1%)	833 (74.8%)	1276 (68.8%)	0.134

All variables were measured at baseline (study entry). Continuous variables are presented as the median (Q1–Q3) and compared using the Wilcoxon rank-sum test. Categorical variables are presented as n (%) and compared using Pearson's chi-squared test. SMD—standardised mean difference; LDL-C—low-density lipoprotein cholesterol; eGFR – estimated glomerular filtration rate; MI—myocardial infarction; CV—cardiovascular.

Table S2. Multivariable Cox PH model demonstrating the association of Lp(a) with all-cause mortality, including sex interaction. .

Variable	HR (95% CI)	p-value
Lp(a) 51–90 mg/dL (vs ≤50)	1.06 (0.85–1.34)	0.592
Lp(a) >90 mg/dL (vs ≤50)	1.04 (0.80–1.34)	0.775
Lp(a) 51–90 × male sex	0.84 (0.62–1.15)	0.277
Lp(a) >90 × male sex	0.81 (0.57–1.15)	0.242
Male sex	1.26 (1.14–1.40)	<0.001
eGFR <60 mL/min/1.73 m^2	1.79 (1.59–2.02)	<0.001
Hypertension	0.83 (0.75–0.92)	<0.001
LDL-C (per 1 mg/dL)	0.92 (0.88–0.96)	<0.001

Reduced kidney function violated the PH assumption and was modelled as a time-dependent covariate. The model includes an interaction term between Lp(a) categories and sex. HRs are adjusted for all shown covariates and interpreted at the same attained age. Reference category for Lp(a): ≤50 mg/dL; reference sex: female. LDL-C—low-density lipoprotein cholesterol; Lp(a) —lipoprotein(a); HR—hazard ratio; PH—proportional hazard.
